# Free-living and captive turtles and tortoises as carriers of new *Chlamydia* spp.

**DOI:** 10.1371/journal.pone.0185407

**Published:** 2017-09-26

**Authors:** Agata Mitura, Krzysztof Niemczuk, Kinga Zaręba, Magdalena Zając, Karine Laroucau, Monika Szymańska-Czerwińska

**Affiliations:** 1 Department of Cattle and Sheep Diseases, National Veterinary Research Institute, Pulawy, Poland; 2 Department of Microbiology, National Veterinary Research Institute, Pulawy, Poland; 3 University Paris-Est, Anses, Animal Health Laboratory, Bacterial Zoonoses Unit, Maisons-Alfort, France; Defense Threat Reduction Agency, UNITED STATES

## Abstract

A variety of *Chlamydia* species belonging to the *Chlamydiaceae* family have been reported in reptilian hosts but scarce data about their occurrence in turtles and tortoises are available. In this study, research was conducted to acquire information on invasive alien species (IAS) of turtles and indigenous turtles and tortoises, living both free and in captivity, as possible reservoirs of *Chlamydiaceae*. Analysis of specimens (pharyngeal and cloacal swabs and tissues) from 204 turtles and tortoises revealed an overall *Chlamydiaceae* prevalence of 18.3% and 28.6% among free-living and captive animals respectively, with variable levels of shedding. Further testing conducted with a species-specific real-time PCR and microarray test was unsuccessful. Subsequently sequencing was applied to genotype the *Chlamydiaceae*-positive samples. Almost the full lengths of the 16S rRNA and *omp*A genes as well as the 16S-23S intergenic spacer (IGS) and 23S rRNA domain I were obtained for 14, 20 and 8 specimens respectively. Phylogenetic analysis of 16S rRNA amplicons revealed two distinct branches. Group 1 (10 specimens), specific to freshwater turtles and reported here for the first time, was most closely related to *Chlamydia* (*C*.*) pneumoniae* strains and the newly described *Candidatus* C. sanzinia. Group 2 (four specimens), detected in *Testudo* spp. samples, showed highest homology to *C*. *pecorum* strains but formed a separate sub-branch. Finally, molecular analysis conducted on positive samples together with their geographical distribution in places distant from each other strongly suggest that Group 1 specimens correspond to a new species in the *Chlamydiaceae* family. In-depth studies of *Chlamydia* spp. from turtles and tortoises are needed to further characterise these atypical strains and address arising questions about their pathogenicity and zoonotic potential.

## Introduction

Chlamydiae are bacteria belonging to the *Chlamydiaceae* family. Their host specificity varies: e.g. *C*. *caviae* is found in guinea pigs, whereas *C*. *pneumoniae*, *C*. *pecorum* or *C*. *abortus* can infect different animals as well as humans. Besides the well-known *Chlamydia* species, new species and genotypes are constantly being discovered in different hosts and environments [1–6]. The first report of a chlamydial agent in a reptile, the eastern fence lizard (*Sceloporus undulatus)*, dates back to 1944 [7]. Since then, *Chlamydiaceae* have been observed among more reptiles including the Burmese python [8, 9], emerald tree boa [10], western sand viper [11], chameleon [12], crocodiles (Nile, Indo-Pacific and Siamese) [5, 13, 14] and tortoises belonging to the *Testudinidae* family [15, 16]. Species determination in *Chlamydiaceae*-positive samples was not always possible but retrospective study and the newest research have shown *C*. *pneumoniae* presence in snakes and chameleon, and other yet unclassified *Chlamydia* spp. in the remaining cases [17]. Recently, candidate species C. sanizinia was reported for the first time in a Madagascar tree boa (*Sanzinia madagascariensis volontany*) [3].

Threats arising from the introduction of invasive alien species (IAS) are well documented in different environments around the globe [18, 19]. Pond sliders (*Trachemys* (*T*.) *scripta*) are turtles native to North America which have been imported in their thousands to European countries in recent years as companion animals. Numerous reports on their introduction to natural environments in different locations, e.g. France, Italy, Switzerland, and Japan have been published and cases of successful reproduction have been confirmed [20, 21]. The pond sliders (including three subspecies, namely the red-eared slider (*T*. *s*. *elegans*), the Cumberland slider (*T*. *s*. *troostii*) and the yellow-bellied slider (*T*. *s*. *scripta*)) together with the common snapping turtle (*Chelydra serpentina*), the painted turtle (*Chrysemys picta*) and the false map turtle (*Graptemys pseudogeographica)* are now listed as reptilian IAS not only in Polish [22] but also in European Union legislation [23, 24]. Negative impact of IAS on biodiversity may manifest in predation or competition with resident species and their often being a reservoir of new viral and/or bacterial pathogens such as the *Chlamydiaceae* or *Enterobacteriaceae* families [25]. The main threat in Poland is posed to the endangered and strictly protected European pond turtle (*Emys* (*E*.) *orbicularis*), the only native freshwater turtle. Significant populations of IAS, mainly *T*. *scripta*, have been observed in south-eastern regions for at least 10 years (B. Gorzkowski, personal communication). These species share an ecological niche with *E*. *orbicularis* and compete for food and basking places. Reptiles can often be found in recreational areas, such as zoos, parks and lakes, and their popularity as pets is ever-increasing nowadays. Taking into account the occurrence of *Chlamydiaceae* in these poikilothermic animals, zoonotic implications should also be considered [9, 11]. Thus, a survey was conducted in Poland to acquire information on both free-living and captive turtle (semi-aquatic animal) and tortoise (land animal) species as possible reservoirs of *Chlamydiaceae*. Positive samples were subjected to further molecular and phylogenetic characterisation.

## Materials and methods

### Project presentation

The present survey is a part of a project titled “Invasive turtle species as a source and vector of animal and human pathogens” started to assess environmental and epidemiological threats posed by IAS turtles and their impact on biodiversity in Poland. This interdisciplinary project included trapping, clinical examination, quarantine, euthanasia and anatomopathological examination.

### Samples

**Fig 1:**
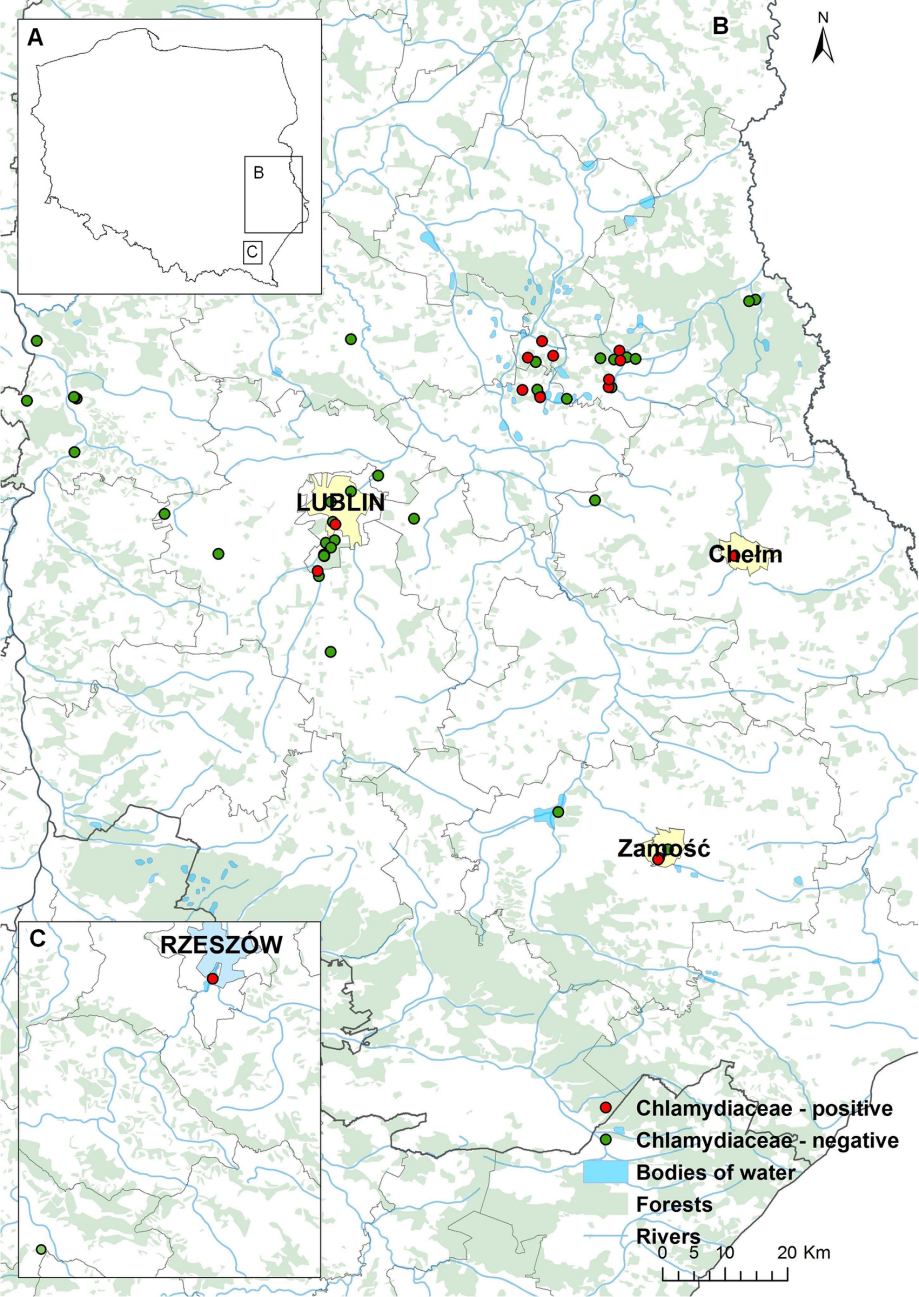
Sampling area of IAS turtles. Lubelskie (B) and Podkarpackie (C) voivodeships are marked with squares on the contour of Poland (A). Sites of animal capture are marked on detailed maps of the areas (ArcMap10.4 software).

Samples were taken from free-living and captive turtles and tortoises from private households or zoo collections during the summers of 2015 and 2016. Cloacal and pharyngeal swabs as well as tissue samples were obtained from free-living, whereas only cloacal swabs were sampled from captive animals. Flocked swabs were used throughout the study. Table 1 summarizes data on results, animal species, free or captive status and type of samples collected. Altogether 204 animals were tested. These included 68 IAS turtles captured in Lubelskie voivodeship, eastern Poland (Fig 1) as well as three adult European pond turtles (*E*. *orbicularis*). These animals were submitted to a cloacal and pharyngeal swabbing (n = 68) during the medical examination at the beginning of quarantine. Seven pond sliders were resampled at the end of this period to follow chlamydiae shedding. Euthanasia was performed with the use of Morbital (Biowet Pulawy, Poland) administered intravenously after at least two weeks of clinical observation and was followed by necropsy. Tissue samples were collected including specimens from the respiratory (n = 58) and reproductive (n = 57) tracts as well as sections from cloacae (n = 46) of 58 animals. The only adult European pond turtle available for necropsy showed signs of starvation and advanced autolysis and its reproductive tract was not sampled. Additionally, 19 European pond turtle hatchlings which had died in a breeding facility and were submitted for testing by Polesie National Park were included in the study. In this instance, all tissues were pooled together due to the scarcity and bad conservation of the material and were tested as a single sample. Tissue homogenates were prepared in saline solution or PBS at 1:2 dilution and 100 μL of homogenate was used for DNA isolation.

**Table 1:** Summary of *Chlamydiaceae*-specific real-time PCR results.

	No of *Chlamydiaceae* positive / No of tested animals (%)	Type of sample				
		positive / tested swabs (%)		positive / tested tissues (%)		
		pharyngeal	cloacal	respiratory tract	reproductive tract	cloacae
**Free-living turtles**						
**Invasive alien species (IAS)**						
Pond slider (*Trachemys scripta*)	13/63* (20.6)	6/60* (10.0)	11/60* (18.3)	3/56 (5.4)	2/56 (3.6)	5/44 (11.4)
Common snapping turtle (*Chelydra serpentina*)	0/1	0/1	0/1	na	na	na
False map turtle (*Graptemys pseudogeographica*)	0/2	0/2	0/2	0/1	0/1	0/1
River cooter (*Pseudemys concinna)*	0/1	0/1	0/1	na	na	na
Florida red-bellied cooter (*Pseudemys nelsoni*)	0/1	0/1	0/1	na	na	na
**Native species**						
European pond turtle (*Emys orbicularis*)	0/3	0/3	0/3	0/1	0/0	0/1
**Total**	13**/71** (18.3)	**6/68** (8.8)	**11/68** (16.2)	**3/58** (5.2)	**2/57** (3.5)	**5/46** (10.9)
European pond turtle (*Emys orbicularis*) (pool of 19)	1/1**	na	na	na	1/1**	na
**Captive turtles/tortoises**						
**Captive turtles**						
Pond slider (*Trachemys scripta*)	12/33 (36.4)	na	12/33 (36.4)	na	na	na
River cooter (*Pseudemys concinna*)	0/1	na	0/1	na	na	na
European pond turtle (*Emys orbicularis*)	0/10	na	0/10	na	na	na
**Captive tortoises**						
Leopard tortoise (*Psammobates pardalis*)	0/1	na	0/1	na	na	na
Radiated tortoise (*Astrochelys radiata*)	0/1	na	0/1	na	na	na
Horsfield's tortoise (*Testudo horsfieldii*)	0/48	na	0/48	na	na	na
Hermann's tortoise (*Testudo hermanni)*	1/2 (50.0)	na	1/2 (50.0)	na	na	na
Marginated tortoise (*Testudo marginata)*	2/2 (100.0)	na	2/2 (100.0)	na	na	na
*Testudo* spp.***	23/35 (65.7)	na	23/35 (71.4)	na	na	na
**Total**	**38/133** (28.6)		**38/133** (28.6)			

* Three deceased animals were included and only tissue samples were tested.

** Pooled sample of all tissues from 19 European pond turtle hatchlings was tested

*** No detailed data on animal species were available.

Additionally, 44 captive turtles and 89 captive tortoises from private households and zoo collections were sampled. Cloacal swabs were collected from these animals during routine activities following standard procedures. All samples were stored at −20°C prior to DNA extraction.

### Ethics statement

According to Polish and European Union legislation, ethical approval is not necessary to capture and euthanize invasive alien species which threaten ecosystems, habitats or species [24, 26]. Ethical approval for animal experiments was also not required according to the Local Ethical Committee on Animal Testing at the University of Life Sciences in Lublin (statement of 14 Oct 2014). The contribution of additional samples delivered by Polesie National Park (letter of 26 Jun 2016) and laboratory testing of samples originating from protected animal species were approved by Regional Directorate for Environmental Protection resolution no. WPN.6401.206.2016MPR.

### DNA extraction

DNA isolation was performed on 100 μL of tissue homogenates, pharyngeal or cloacal swabs with a QIAamp DNA Mini Kit (Qiagen, Germany) according to the manufacturer’s protocol, with a final elution volume of 200 μL. DNA was aliquoted and stored at −20°C before use.

### Molecular analysis

#### Real-time PCR

A real-time PCR according to Ehricht et al. [27] was used as a screening method with primers specific for the *Chlamydiaceae* family. All primers used in the study are listed in S1 Table. The positive control for PCR, *C*. *trachomatis* (Genekam, Germany) and DNase-RNase free water (Qiagen, Germany) as the negative control were included in each run. To ensure that no inhibition was observed, an internal amplification control (TaqMan Exogenous Internal Positive Control, Applied Biosystems, USA) was added to all analysed samples according to the manufacturer’s protocol. An analytical cut-off value of 38 was selected corresponding to the defined lower limit of detection of the test. Any Ct values above this defined limit were thereafter considered unreliable.

#### Species designation

All *Chlamydiaceae*-positive samples were re-tested with real-time PCRs specific for *C*. *pneumoniae* and *C*. *pecorum*. Primers and probe sets according to Kohlhepp et al. [28] and Pantchev et al. [29] were used.

#### Microarray

*Chlamydiaceae*-positive samples from 10 arbitrarily selected animals from among those with the lowest Ct values (Ct<34) were sent to Alere Technologies GmbH (Germany) and subjected to microarray testing.

#### DNA sequencing

Amplification of outer membrane protein A (*omp*A) gene fragments was performed using the primer pair CTU/CTL [30]. The ribosomal RNA locus represented by the almost full lengths of the 16S rRNA gene, 16S-23S intergenic spacer (IGS) and 23S rRNA domain I was amplified by two separate PCR runs as previously described [31–33]. Electrophoretic separation was conducted on 1% agarose gel stained with SimplySafe dye (EurX, Poland). PCR products were sent for sequencing to Genomed (Poland). Sequences were deposited to the GenBank (NCBI) database under accession numbers KY928236–KY928255 (*omp*A), KY928256–KY928270 (16S rRNA), and KY928271–KY928278 (23S rRNA).

### Phylogenetic analysis

Sequencing data were subjected to a BLAST search against the NCBI database to identify the most similar entries and aligned with a set of selected sequences representative for known chlamydial species. To assess the phylogenetic relationship between *Chlamydia* spp. and the tested samples, phylogenetic trees were constructed for 16S rRNA (1180bp) and IGS-23S rRNA (920 bp) as well as for *omp*A (920 bp) using Geneious Pro 8.0 software (Biomatters, New Zealand). The MrBayes method with 1,000 replicates bootstrap was applied. The general time-reversible (GTR) model of DNA substitution was chosen for analysis of the 16S rRNA gene and Hasegawa–Kishono-–Yano (HKY85) for 23S rRNA and *omp*A phylogeny, based on MEGA 6 calculations. An estimation of mean nucleotide distances was prepared with the MEGA 6 software package [34].

## Results

### *Chlamydiaceae* detection and level of shedding

The results of *Chlamydiaceae*-specific real-time PCR in 11 different turtle and tortoise species included in the survey are presented in Table 1 and S2 Table. Examination of samples from 204 animals gave positive results in 51 cases (25.0%). European pond turtles from a breeding facility were also found positive. Cloacal swab specimens collected from turtles and tortoises kept in captivity gave prevalence of 28.6% with 38 positive results. Samples from 48 Horsfield's tortoises kept together were all negative whereas both marginated tortoises and one Hermann's tortoise acquired from different private owners were positive. The presence of *Chlamydiaceae* was detected in 65.7% (23 out of 35) of *Testudo* spp. specimens based on the qPCR test. Prevalence of 36.4% was noted among captive *T*. *scripta*, most of which came from one collection and had direct contact with each other. Among 71 free-living animals 13 (18.3%) were positive in at least one type of sample. Different ways of shedding were observed in IAS turtles. Chlamydial DNA was detected in both pharyngeal and cloacal swabs in four pond sliders, only in cloacal swab in seven turtles and exclusively in pharyngeal swab in a further two animals. In seven animals positive at the first sampling for the cloacal swab, cloacal tissue was confirmed positive for five specimens.

Variable levels of pharyngeal and cloacal shedding were noted in the course of the study (Table 2). Seven pond sliders were sampled again at the end of quarantine exhibiting different loads of chlamydial DNA in swab specimens. An increase in pathogen shedding was observed in two animals (nos. 118 and 119) and a maintained level in one (no. 231) at both sampling sites for two time points. A decrease was shown in two cases (nos. 117 and 211), one of which was connected with the animal becoming negative in specimens tested at the second sampling point. However, examination of a further two *T*. *scripta* (nos. 116 and 237) at the end of quarantine revealed that chlamydia shedding had stopped in cloacae but had started in the upper respiratory tract. Attempts were also made to confirm if only one *Chlamydia* strain was present in the animal when multiple samples returned positive results. Thus, *omp*A sequences were acquired from samples 117A03 (cloacal swab) and 117X83 (respiratory tract homogenate) as well as 213X25 (cloacae tissue) and 213X34 (reproductive system homogenate). Resulting sequences were identical for each of the animals, supporting assumption of a single strain colonization.

**Table 2:** Shedding levels at the beginning and end of quarantine.

Animal no	Pharyngeal swab (Ct)		Cloacal swab (Ct)	
	Time point			
	1	2	1	2
**116**	-	30.5	36.5	-
**117**	28.6	32.4	25.3	32.8
**118**	-	33.7	36.7	31.6
**119**	37.3	32.6	-	31.8
**211**	-	-	35.3	-
**231**	36.6	35.3	36.6	35.1
**237**	-	34.0	38.0	-

Chlamydiae were detected in three out of 58 tissue samples from the respiratory tract (animals nos. 103, 117 and 213) as well as in two samples from the reproductive system (animals nos. 110 and 213). *T*. *scripta* no. 213 was the only one with all specimens positive in the *Chlamydiaceae*-23S assay. The pooled sample of *E*. *orbicularis* hatchlings (specimen no. 153) tested positive while three adult European pond turtles which were available for sampling were not carrying *Chlamydia* spp.

### Genotyping and phylogenetic analysis

**Fig 2:**
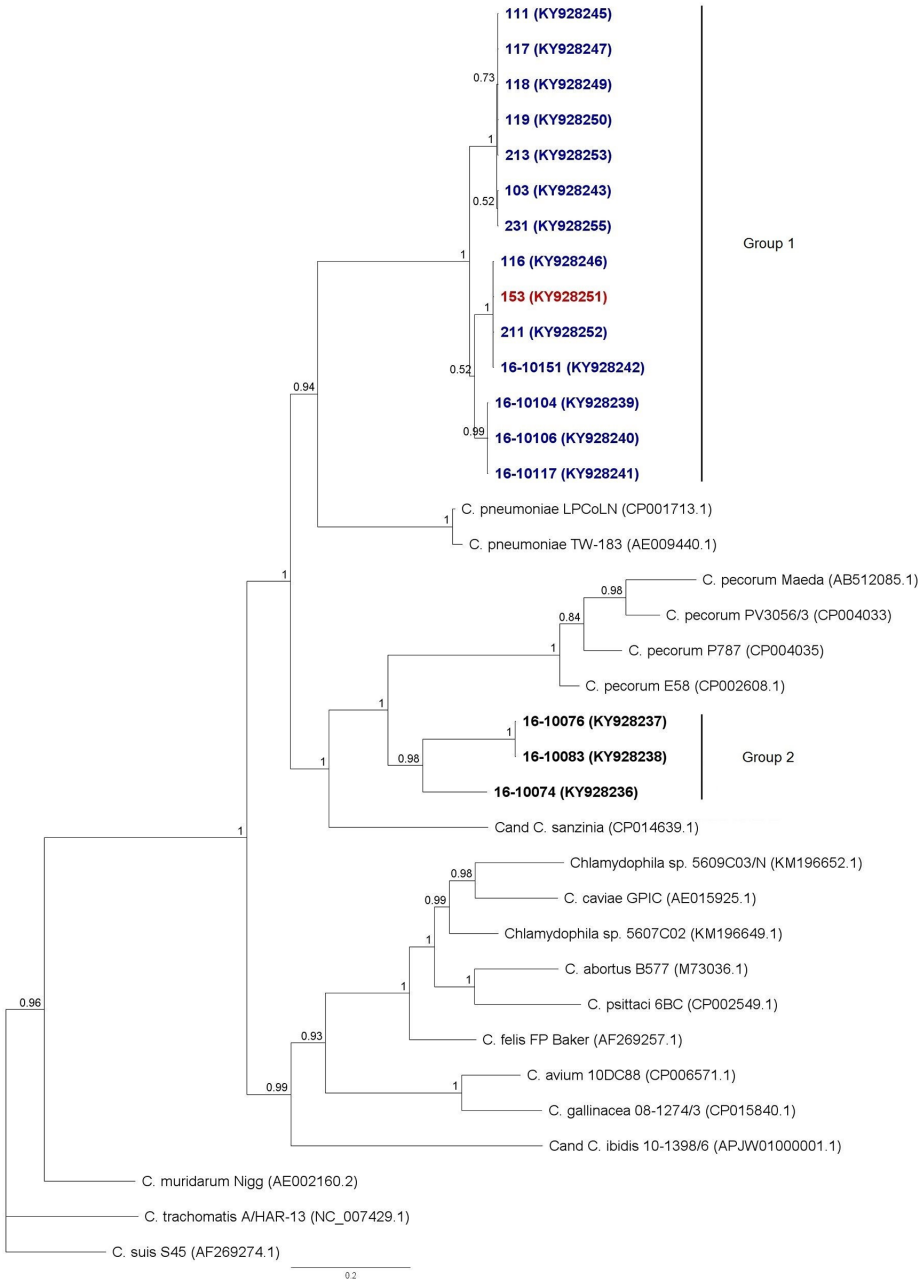
*omp*A-based tree (920bp) showing phylogenetic relationships within *Chlamydiaceae* family. *C*. *trachomatis* strain A/HAR-13 was used as an outgroup. Bayesian inference with a 1000 replicates bootstrap was applied. Sequences obtained in this study are in bold: IAS turtles are marked blue, *E*. *orbicularis* red and tortoises were left black; the bar corresponds to number of substitutions per site.

Microarray testing revealed that two out of 10 randomly selected *Chlamydiaceae*-positive samples (S2 Table) gave positive signals with both *Protochlamydia amoebophila* and *Protochlamydia naegleriophila* probes in microarrays but in the remaining eight cases results were negative. The remaining positive samples were not included in the microarray testing. No positive results were obtained with species-specific PCRs. Thus, sequencing of the *omp*A gene and ribosomal loci was performed to carry on further molecular characterisation of specimens (S2 Table). Partial *omp*A amplicons for 17 animals were obtained and aligned with sequences of *Chlamydiaceae* reference strains and others of reptilian origin. All sequences from the current study precisely overlie sequences within the *Chlamydiaceae* family and two distinct and clearly separated branches can be observed as shown on the constructed dendrogram (Fig 2). Group 1 includes sequences from freshwater turtles (pond sliders and European pond turtle hatchlings) and is divided into three sub-branches. One (7 amplicons from *T*. *scripta* samples KY928243, KY928245, KY928247, KY928249, KY928250, KY928253 and KY928255) shows minor heterogeneity (1 SNP) whereas the remaining two are formed by identical sequences from four (KY928242, KY928246, KY928251 and KY928252) and three animals (KY928239 to KY928241) respectively. In all three cases, the maximum posterior probability value supports branch division. All amplicons in the first sub-branch were of free-living pond sliders, the second also contained a captive animal specimen, and sequences incorporated in the last sub-group were all obtained from captive *T*. *scripta* from one collection. The second main branch, namely Group 2, includes three sequences (KY928236 to KY92838) acquired exclusively from *Testudo* spp. samples. Maximum support was calculated for this group and considerable variability can be noted. Short sequences (380 bp) obtained earlier from nasal lavage fluid of *T*. *marginata* (AY845422.1) and *T*. *horsfieldii* (AY845419.1 and AY845423.1) by Hotzel et al. share between 97.2% and 98.3% homology with corresponding sequences 16–10076 and 16–10083 from the current study.

**Fig 3:**
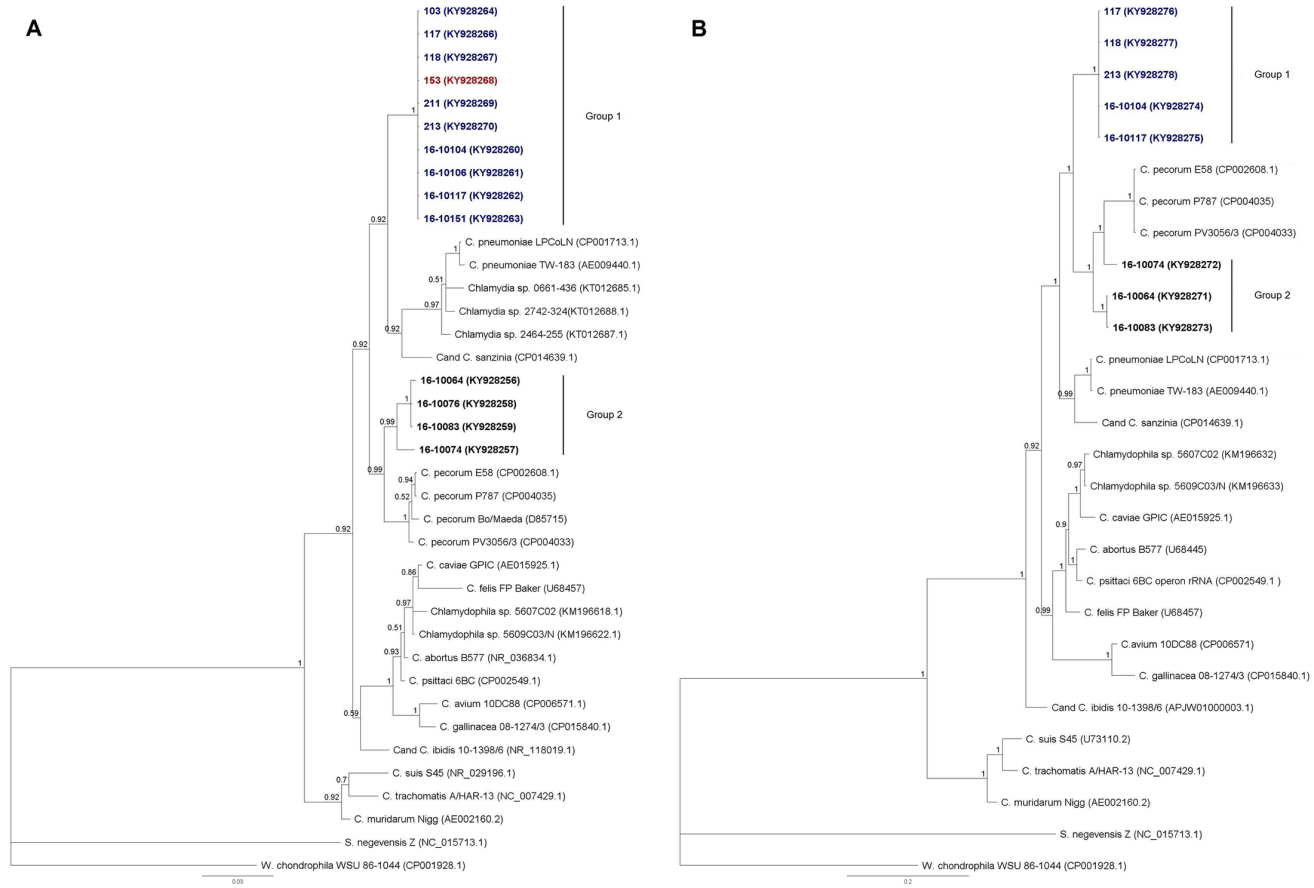
Phylogenetic tree based on 16S rRNA gene fragment (1180bp) (A) and 16S-23S intergenic spacer and nearly full length of 23S rRNA domain I fragment (920bp) (B). Representative sequences of established *Chlamydiaceae* species as well as strains encountered in reptilian hosts were included. *S*. *negevensis* strain Z was used as an outgroup. Bayesian inference with a 1000 replicates bootstrap was applied. Sequences obtained in this study are in bold: IAS turtles are marked blue, *E*. *orbicularis* red, and tortoises were left black; bar corresponds to number of substitutions per site.

Sequencing of the nearly full-length 16S rRNA gene was implemented to designate the chlamydiae species. Fourteen amplicons out of 52 positive animals were acquired, successfully sequenced and used for phylogenetic comparison. Sequences from this study were shown to have up to 38 SNPs in a 1180bp fragment of ribosomal loci. BLAST analysis carried out against the GenBank database revealed 96.8% to 97.8% nucleotide homology to *C*. *pecorum* strains E58 (NR102975.1) and P787 (CP004035.1) and to the *Cand*. C. sanzinia strain 2742–308 (CP014639.1). The phylogenetic tree constructed on the basis of the available 16S rRNA fragments is presented in Fig 3A and shows topology almost identical to the one obtained for the *omp*A gene. Group 1 is monophyletic and gathers 10 sequences (KY928260 to KY928270) from both free-living and captive *T*. *scripta* and *E*. *orbicularis* hatchlings and is supported by a posterior probability value of 1. The remaining four amplicons (Group 2, KY928256 to KY928259) obtained from captive *Testudo* spp. are more heterogeneous and show the highest homology to *C*. *pecorum* sequences, with up to 27 base pair differences to the Maeda strain (D85715). Posterior probability of 0.88 was calculated for this branch. The genetic distances (S3 Table) of each group described in this study to other *Chlamydiales* are noticeably larger (0.202–0.254) than those to members of the *Chlamydiaceae* family (0.028–0.70). Analysis of domain I of the 23S rRNA gene and IGS sequence (Fig 3B) displays a similar pattern to that of 16S rRNA with some differences in branch ordering, though only eight sequences were available.

## Discussion

Threats arising from IAS are well documented in different environments. The knowledge of invaders’ presence in new habitats, population range, behaviour and carried microflora is important in management and eradication strategies [35]. Thus, this study on *Chlamydiaceae* dissemination among free-living reptiles captured in lakes and rivers of eastern Poland was performed. The investigation yielded a high prevalence rate of 18.3%. There are no sufficient data available on *Chlamydia* occurrence in IAS turtles and most published research works concerning reptiles include animals living in captivity or on farms, zoos or in private collections [5, 11, 14, 15, 36]. In our study 28.6% of captive turtles and tortoises were found positive in *Chlamydiaceae*-specific real-time PCR. What is worth noting is that 48 Horsfield's tortoises from a zoo collection did not carry *Chlamydiae* while prevalence in the *T*. *scripta* group kept altogether reached 36.4% and for *Testudo* spp. was even 65.7%. Similar results were obtained by Frutos et al. reporting a prevalence of 57.9% among reptiles in a recreational park in a central area of Argentina [9]. Contrastingly, in a German study on companion animals of the family *Testudinidae* only 10.3% of cases were found to be *Chlamydiaceae*-positive [15]. The high prevalence noted both in our and the Argentinean survey could be due to a shared environment and potential pathogen transfer through personnel tending the animals in tested collections, which was not the case for animals kept separately in private households.

*Chlamydiaceae* in *T*. *scripta* were shed intermittently as shown in Table 2, like other species of this family, e.g. *C*. *psittaci* [37]. Also positive results mainly obtained for swab specimens without simultaneous detection in tissue samples can prove them to be commensals or conditionally pathogenic rather than pathogenic microflora, which was suggested by Hotzel et al. [15]. A decrease in animal immune response caused by temperature changes, stress, malnutrition or other pathogens [38, 39] combined with *Chlamydia* colonization may trigger systemic infection. In our study, chlamydial DNA was detected in all collected samples only for pond slider no. 213. This animal was caught in a small water basin without a basking place or way out and died on the third day of quarantine. Poor overall condition with signs of exhaustion was noted during its clinical examination (N. Chlebicka, personal communication). *Klebsiella* spp. was found in swabs and respiratory tract specimens (A. Kędrak-Jabłońska, personal communication). Moreover, *Mycobacterium* sp. was identified in internal organ samples but no histopathological changes were observed in the liver (M. Lipiec, personal communication). Severe conjunctivitis, which is a typical manifestation of most *Chlamydia* spp. infections, was observed in *T*. *scripta* no. 213 accompanied by accumulation of fibrin (N. Chlebicka, personal communication). Similar signs of chlamydiae presence were also reported by other authors in crocodiles [14, 40] and tortoises [41]. The symptoms described imply that a chlamydial agent is at least one of the components of the disease cause.

High mortality of juvenile European pond turtles was observed at the beginning of 2016 in a breeding facility at Polesie National Park. As an outcome of the investigation into the cause, *Chlamydiaceae* DNA was detected in animal no. 211 (*T*. *scripta*), which was a resident of this facility and was handled by the same personnel as handled freshly hatched European pond turtles. No clinical signs of infection were observed in the pond slider, whereas numerous hatchling deaths were recorded (specimen no. 153). Laboratory testing for *Salmonella*, *Yersinia* and *Mycobacterium* spp. gave negative results (W. Iwaniak and M. Lipiec, personal communication) while the Ct value in *Chlamydiaceae*-23S real-time PCR was 28.7, indicating high bacterial load in tissues. Sequences of the *omp*A gene (950 bp) from animal 211’s (*T*. *scripta*) and 153’s (*E*. *orbicularis* hatchlings) specimens were aligned and confirmed to be identical. These data could point to transfer of chlamydiae from *T*. *scripta* to *E*. *orbicularis* hatchlings. The immaturity of the immune system in juveniles or a possible concurrent infection (e.g. viral) could result in the systemic spread of *Chlamydiae* and a fatal outcome. A similar case was reported by Huchzermeyer et al. in juvenile crocodiles [13]. Uncorroboratively, none of the 13 adult European pond turtles, neither free-living nor captive originating from different locations, tested positive. Detailed research is needed to acquire information about whether *E*. *orbicularis* local populations are harbouring *Chlamydia* spp. or if these bacteria carried by IAS turtles can further endanger protected species.

*C*. *pneumoniae* and *C*. *psittaci* are the chlamydial species most often encountered in reptiles [8, 9, 11, 42]. Research by Kabeya et al. on zoo animals in Japan showed a significantly higher prevalence of *Chlamydia* in reptiles than in birds and mammals as well as the dominance of *C*. *psittaci* over *C*. *pneumoniae*. However, Sariya et al. [5] confirmed the presence of *Chlamydia* sp. most closely related to *C*. *caviae* in Siamese crocodiles (*Crocodylus siamensis*) on farms in Thailand. The results of the current study confirm that the variety of *Chlamydia* spp. encountered in reptilian hosts is even broader. Phylogenetic analysis of ribosomal gene sequences from these specimens allowed the distinction of two genotypes. Group 1 seems to be specific to semi-aquatic species, namely *T*. *scripta* and *E*. *orbicularis*. To date, no related sequence from other hosts has been found in GenBank. The 16S rRNA homologies of these sequences with *C*. *pecorum* E58 and *Cand*. C. sanzinia are 96.8% and 97.4% respectively. Moreover Group 1 clearly branches away from both mentioned species. In support of these data, 23S rRNA sequences as well as the distribution of this group of specimens in geographically separated places also suggest their classification as new species in the *Chlamydiaceae* family.

Group 2 on the other hand is most closely related to *C*. *pecorum* strains but still forms a separate sub-branch. All specimens included in this group were detected in *Testudo* spp. These data are in accordance with previous results published by Hotzel and colleagues [15]. Amplicons from our survey belong to a branch displaying the same phylogenetic position on the 16S rRNA and *omp*A dendrograms and reinforce assignment of the agent, as suggested in previous publications, to a new species with tortoises as hosts. Two sequences, namely 16–10076 and 16–10083, share 100% similarity across 214 bp of the 16S rRNA signature region with sequence AY845424 from a previous report, although corresponding *omp*A fragments display notable variation [15].

Although available phylogenetic data are sufficient for preliminary species recognition, in both cases further work is needed for an extensive characterisation of turtle and tortoise-specific *Chlamydiaceae*. Attempts were made at strain isolation but no successful growth was observed. Difficulties in culture may be due to the poikilothermic character of reptiles influencing the microbiome of these animals and the requirements of strongly adapted bacteria. Thus, adjustments of standard isolation procedures used for chlamydial strains infecting higher vertebrates have to be made.

Many earlier reports draw attention to zoonotic risks connected with reptiles kept as companion animals. *Salmonella* spp. [43, 44] and *Mycobacterium* spp. [45] were found in captive animals sometimes representing a serious hazard for people’s health [46–48]. It can also be the case with *Chlamydia* spp., as has already been suggested, especially when *C*. *pneumoniae* and *C*. *psittaci* were noted. Nonetheless our knowledge of newly described species is insufficient to establish their zoonotic implications, which still cannot be excluded. The numbers of reptiles imported to Poland in recent years are overwhelming and a considerable part of them might at some point be freed into the wild, joining the IAS population and consequently disturbing the local biodiversity. At the same time they can pose a direct threat to the public, as lakes and rivers often used as recreational zones are usually chosen for the release of the turtles.

Our research revealed the presence of two different chlamydial agents in turtles and tortoises with a considerable level of shedding for some of them. Phylogenetic analysis showed that even though a variety of *Chlamydia* spp. was already reported in reptiles, Group 1 encountered in semi-aquatic turtles is reported here for the first time. It is not known if the *Chlamydiaceae* described in our research was already present in the population of the endangered native species *E*. *orbicularis* or otherwise if it was transferred from an alien species of *T*. *scripta*. Moreover, no detailed data on the microbiome of IAS turtles is available, thus the zoonotic character of more pathogens than only chlamydial species has to be kept in mind. Further studies are needed to expand our knowledge on all presented issues.

## Supporting information

S1 Table Summary of primers used in the study. (DOCX)

S2 Table Sample identity and origin with summary of tests results. (XLSX)

S3 Table Mean nucleotide distances calculated for 1180bp fragment of 16S rRNA gene among isolates obtained in this study, species of *Chlamydiaceae*, *Simkaniaceae* and *Wadlliaceae* families as well as *Chlamydia* spp. from other reptilian hosts. (XLS)
