# Supplementary material for: Free-living and captive turtles and tortoises as carriers of new Chlamydia spp
Source: PLoS One. 2017 Sep 26;12(9):e0185407. doi: 10.1371/journal.pone.0185407 (PMC5614609; doi:10.1371/journal.pone.0185407)
Supplement: S1 Table — (DOCX) [file pone.0185407.s001.docx]

|  | **Primer and probes** | **Sequence (5'-3')** | **Target gen** | **Reference** |
| --- | --- | --- | --- | --- |
| **Real-time PCRs** |  |  |  |  |
| *Chlamydiaceae*-23S |  |  | 23S rRNA | Ehricht et al. 2006[1] |
|  | Ch23S-F | CTGAAACCAGTAGCTTATAAGCGGT |  |  |
|  | Ch23S-R | ACCTCGCCGTTTAACTTAACTCC |  |  |
|  | Ch23S-p | FAM-CTCATCATGCAAAAGGCACGCCG-TAMRA |  |  |
| *C. pneumoniae* |  |  | ompA | Kohlhepp et al. 2005[2] |
|  | Forward | AAGGGCTATAAAGGCGTTGCT |  |  |
|  | Reverse | AGACTTTGTTCCAGTAGCTGTTGCT |  |  |
|  | Probe | TCCCCTTGCCAACAGACGCTGG |  |  |
| *C. pecorum* |  |  | ompA | Pantchev et al. 2010[3] |
|  | CppecOMP1-F | CCATGTGATCCTTGCGCTACT |  |  |
|  | CppecOMP1-R | TGTCGAAAACATAATCTCCGTAAAAT |  |  |
|  | CppecOMP1-S | FAM-TGCGACGCGATTAGCTTACGCGTAG-TAMRA |  |  |
| **Sequencing** |  |  |  |  |
|  | CTU | ATGAAAAAACTCTTGAAATCGG | ompA, ~1000bp | \| Denamur et al. 1991[4] \| \| --- \| \| |
|  | CTL | CAAGATTTTCTAGA(T/C)TTCAT(C/T)TTG |  |  |
|  | 16S1 | CGGATCCTGAGAATTTGATC | 16S rRNA, ~1400bp | Pudjiatkomo et al. 1997[5]; Thomas et al. 2006[6] |
|  | rp2 | CTACCTTGTTACGACTTCAT |  |  |
|  | 16SF2 | CCGCCCGTCACATCATGG | intergenic spacer, 23S rRNA, ~1000bp | Everett et al. 1999[7] |
|  | 23SIGR | TGGCTCATCATGCAAAAGGCA |  |  |

1. Ehricht R, Slickers P, Goellner S, Hotzel H, Sachse K. Optimized DNA microarray assay allows detection and genotyping of single PCR-amplifiable target copies. Mol Cell Probes. 2006;20: 60–63. doi:DOI 10.1016/j.mcp.2005.09.003

2. Kohlhepp SJ, Hardick J, Gaydos C. *Chlamydia pneumoniae* in peripheral blood mononuclear cells isolated from individuals younger than 20 years or older than 60 years. J Clin Microbiol. 2005/06/16. 2005;43: 3030. doi:43/6/3030 [pii] 10.1128/JCM.43.6.3030.2005

3. Pantchev A, Sting R, Bauerfeind R, Tyczka J, Sachse K. Detection of all *Chlamydophila* and *Chlamydia* spp. of veterinary interest using species-specific real-time PCR assays. Comp Immunol Microbiol Infect Dis. 2010;33: 473–484. doi:DOI 10.1016/j.cimid.2009.08.002

4. Denamur E, Sayada C, Souriau A, Orfila J, Rodolakis A, Elion ’ J. Restriction pattern of the major outer-membrane protein gene provides evidence for a homogeneous invasive group among ruminant isolates of *Chlamydia* *psittaci*. J Gen Microbiol. 1991;137: 2525–2530.

5. Pudjiatmoko Fukushi H, Ochiai Y, Yamaguchi T, Hirai K. Phylogenetic analysis of the genus C*hlamydia* based on 16S rRNA gene sequences. Int J Syst Bacteriol. 1997;47: 425–431. doi:10.1099/00207713-47-2-425

6. Thomas V, Casson N, Greub G. *Criblamydia* *sequanensis*, a new intracellular *Chlamydiales* isolated from Seine river water using amoebal co-culture. Environ Microbiol. 2006;8: 2125–2135. doi:10.1111/j.1462-2920.2006.01094.x

7. Everett KD, Bush RM, Andersen AA. Emended description of the order *Chlamydiales*, proposal of *Parachlamydiaceae* fam. nov. and *Simkaniaceae* fam. nov., each containing one monotypic genus, revised taxonomy of the family *Chlamydiaceae*, including a new genus and five new species, and standards for the identification of organisms. Int J Syst Bacteriol. 1999;49: 415–440.
